# Self‐Assembled Skin Equivalents with Monoclonal CRISPR/Cas9‐Modified N/TERT‐1 Keratinocytes: A Cutting‐Edge Model for Human Skin and its Diseases

**DOI:** 10.1002/adhm.71283

**Published:** 2026-05-28

**Authors:** Marta Slaufova, Tugay Karakaya, Michela Di Filippo, Thomas Kündig, Hans‐Dietmar Beer

**Affiliations:** ^1^ Department of Dermatology University Hospital Zurich Schlieren Switzerland; ^2^ Faculty of Medicine Department of Dermatology University of Zurich Zurich Switzerland

**Keywords:** crispr/Cas9, monoclonal knockout, N/TERT‐1 keratinocytes, scaffold‐free, skin equivalent

## Abstract

Human skin is a complex organ consisting of multiple cell types and serves as an essential barrier against environmental stressors. Due to ethical considerations and interspecies differences, in vitro human skin equivalents (SEs) are increasingly used to complement or replace animal models in mechanistic, pharmacological, and disease‐modeling studies. Scaffold‐free full‐thickness SEs, in which fibroblasts generate their own extracellular matrix, are particularly attractive because they provide high structural stability even during extended culture. However, the use of genetically defined keratinocyte populations in these SEs has remained limited. Here, scaffold‐free full‐thickness SEs incorporating wild‐type, polyclonal or monoclonal CRISPR/Cas9‐modified N/TERT‐1 keratinocytes, generated via electroporation, are established. Monoclonal N/TERT‐1 keratinocytes with targeted knockout (KO) of the crucial inflammasome component apoptosis‐associated speck‐like protein containing a caspase recruitment domain (ASC) form a differentiated epidermis but fail to secrete the proinflammatory cytokines interleukin (IL)‐1β and IL‐18 upon inflammasome activation, indicating complete functional ablation of inflammasome signaling in the 3D model. Moreover, SEs generated with gasdermin A (GSDMA)‐KO N/TERT‐1 keratinocytes illustrate the feasibility of analyzing genes induced during keratinocyte differentiation under physiological conditions. These results establish scaffold‐free full‐thickness SEs with monoclonal genetically modified N/TERT‐1 keratinocytes as a robust and reproducible human skin model for mechanistic studies and future disease‐modeling applications.

## Graphical abstract

Self‐assembled, scaffold‐free full‐thickness skin equivalents with monoclonal, genetically modified N/TERT‐1 keratinocytes represent a novel in vitro model of human skin and skin diseases. The model is highly robust, reproducible, physiologically relevant, and suitable for high‐throughput applications. By closely mimicking human skin structure, it enables gene function studies under 3D in vivo‐like conditions.

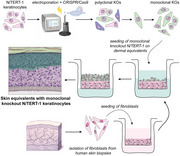

## 1 Introduction

The skin and its appendages represent the physical and immunological barrier of the body, protecting against environmental stressors such as UV radiation, pathogens, and injury [1, 2].

Its outermost compartment, the epidermis, is a stratified epithelium mainly comprised of keratinocytes at distinct stages of differentiation [1]. It is a constantly renewing tissue, where, under homeostatic conditions, proliferation is restricted to the inner basal cell layer, which harbors stem cells and transit amplifying cells. From there, keratinocytes migrate outward, change their expression pattern, and eventually form the outermost keratinized surface layer called the *stratum corneum*, consisting of dead keratinocytes and populated by a characteristic commensal microbiome [3]. In contrast, the underlying dermis is a connective tissue with a fibroblast‐derived extracellular matrix (ECM), where the skin's appendages are embedded, and blood and lymphatic vessels are located. The two compartments are separated by a specialized ECM structure, the basement membrane. Furthermore, the epidermis, and particularly the dermis, harbor additional cell types, including immune cells [2].

Skin models are required to study skin biology in health and disease, to investigate responses to environmental stressors, and to support the development of therapeutic and cosmetic applications [4, 5, 6, 7]. For decades, animal models, particularly rodents, have been widely used in basic and translational skin research due to their systemic complexity and genetic tractability [8, 9]. Genetic engineering of the murine genome in a tissue‐specific manner enables investigation of the cutaneous functions of specific genes or gene mutants during development, homeostasis, and disease. However, significant anatomical, physiological, and molecular inter‐species differences limit the translatability of results obtained from murine skin models to human skin [5]. For example, murine skin is significantly thinner and less stratified than human skin, and is instead covered and protected by hair [10]. The identity between murine and human genes predominantly expressed in the skin is only 30.2%, demonstrating substantial inter‐species differences even at the molecular level [11]. In addition, high costs, ethical concerns, and regulatory restrictions, such as the European ban on animal testing for cosmetic products, have further driven the development of human‐based in vitro skin models [5, 12, 13].

Human skin models are increasingly filling the gap that has opened with the decline in animal experimentation [4, 5, 6, 7, 14, 15]. The most frequently used systems in skin research include two‐dimensional (2D) monocultures or co‐cultures of fibroblasts, typically human primary fibroblasts (HPFs), and keratinocyte cell lines. The isolation of human primary keratinocytes (HPKs) from human surplus skin and their in vitro cultivation was established decades ago and is based on a “feeder cell” technique with mitotically inactivated fibroblasts, which already points to an important crosstalk between fibroblasts and HPKs, which is particularly critical for the latter [16, 17, 18]. Keratinocyte cell lines such as HaCaT [19], generated by spontaneous immortalization, or the well‐characterized, and mainly diploid N/TERT [20], represent alternatives to HPKs, because they retain their proliferative capacity for long‐term culture and avoid the donor‐specific variability associated with primary cells.

Although widely used, 2D cultures of fibroblasts and keratinocytes do not recapitulate the epidermal three‐dimensional (3D) architecture of human skin. Upon exposure to the air‐liquid interface, HPKs, as well as N/TERT‐1 and N/TERT‐2G keratinocytes, but not, for example, HaCaT keratinocytes, can form a 3D stratified epidermis with a cornified layer, termed an epidermal equivalent (EE) [21, 22, 23]. EEs resemble human epidermis in various aspects and even allow microbial colonization for analysis of host‐microbiome interactions [24]. Full‐thickness skin equivalents (SEs) represent the next level of complexity of 3D skin models, combining a dermal equivalent (DE) containing HPFs with an overlying keratinocyte layer that stratifies at the air–liquid interface [25]. These models enable characterization of the crosstalk between fibroblasts and keratinocytes, as both cell types are required for proper differentiation of HPKs and are essential for the formation of a basement membrane [5]. Most SEs are based on purified collagen (often of animal origin) as a scaffold into which HPFs can be incorporated during the scaffold polymerization, although alternative materials such as fibrin‐ or hyaluronic acid‐based matrices and decellularized dermal scaffolds have also been used [7, 26, 27, 28]. Commercially available SEs are based on this approach, and typically contain HPKs in the epidermal compartment [4, 5, 6, 29]. In scaffold‐free full‐thickness SEs, HPFs produce their own ECM, resulting in a dermal compartment that more closely resembles native dermal architecture compared to scaffold‐based SEs. This approach has evolved from an initial sheet‐based strategy, in which fibroblasts were cultured in the presence of ascorbic acid to generate ECM‐rich stromal sheets that were subsequently stacked to reconstruct the dermal compartment [30]. More recent approaches generate fibroblast‐derived matrix DEs by repeated fibroblast seeding directly on cell culture inserts and prolonged in situ matrix deposition, yielding a stable dermal compartment that supports long‐term epidermal regeneration [31]. When combined with keratinocytes, the latter form a well‐stratified epidermis with functional barrier properties [4]. In scaffold‐based SEs, fibroblast‐mediated contraction of the collagen matrix is a frequent event and a major limitation [5]. It was shown that fibroblasts can condense hydrated collagen lattices into tissue‐like structures, establishing contraction as a fundamental property of fibroblast–matrix interactions [32]. Moreover, fibrin‐derived matrices used in skin bioengineering are likewise susceptible to marked contraction [33]. This phenomenon is significantly reduced in self‐assembled SEs, which also support extended culture periods and sustained epidermal regeneration, indicating a more stable tissue equilibrium [6, 14, 31].

More recently, novel dynamic skin culture approaches such as bioreactors, skin‐on‐a‐chip systems, or 3D bioprinting have been developed to mimic circulation or mechanical stimulation of the skin, however, up to now, they lack the well‐established epidermal stratification and cornification achieved in SEs [4, 34]. Furthermore, SEs have been complemented with additional cell types present in human skin, including melanocytes and immune cells, as well as vascular and neural components, highlighting the versatility of this approach [34, 35, 36, 37, 38].

For addressing the roles of specific proteins or for establishing genetic skin disease models, keratinocytes (or/and fibroblasts) can be genetically modified prior to incorporation into SEs. Due to its simplicity, efficiency, and specificity, the CRISPR/Cas9 technique has become the method of choice for genome editing. The generation of knockout (KO) cells is typically based on the non‐homologous end joining (NHEJ) DNA repair pathway, whereas homology‐directed repair (HDR) can be used for precise modifications, including correction of disease‐associated mutations in HPKs [39, 40]. Polyclonal CRISPR/Cas9‐modified keratinocytes have previously been used in organotypic and reconstructed 3D skin models to study gene function and disease mechanisms [41, 42]. To enable the isolation and expansion of single‐cell clones after editing, keratinocyte cell lines are commonly used because of their unlimited proliferation capacity. In contrast, HPKs have a limited replicative lifespan, which makes clonal expansion more challenging. Delivery of CRISPR/Cas9 components has evolved from viral transduction, which results in sustained expression of Cas9 and guide RNA (gRNA), to electroporation of ribonucleoprotein (RNP) complexes [43, 44, 45]. Protocols for the generation of KOs in HPKs based on lentiviral transduction are available, but are associated with toxic and unwanted immunological effects and, due to permanent expression of gRNA and Cas9 protein, with a risk of unspecific targeting effects, especially over time [46, 47]. In contrast, electroporation has proven to be an excellent technique for the delivery of gRNA and Cas9 protein complexes, leading to the generation of efficient and specific KOs in keratinocytes [40, 43, 44, 45, 48]. Based on this approach, *FLG* ^−/−^ N/TERT keratinocytes were generated and their culture in EEs represents a model for atopic dermatitis or ichthyosis vulgaris [49]. Ground‐breaking examples for disease models using scaffold‐based SEs are harlequin ichthyosis or Netherton syndrome with *ABCA12* ^−/−^ N/TERT‐1 or *SPINK5* ^−/−^ HPKs, respectively [48, 50]. These studies illustrate the utility of edited keratinocytes in 3D epidermal or skin‐equivalent systems, but they have largely relied on EEs or scaffold‐based full‐thickness models rather than scaffold‐free full‐thickness SEs.

Here, conditions were established under which N/TERT‐1 keratinocytes can be cultured in scaffold‐free SEs. SEs generated with mono‐ or polyclonal CRISPR/Cas9‐based N/TERT‐1 keratinocytes, produced by electroporation of RNP complexes, enable the investigation of gene function in a physiological human skin model and the establishment of skin disease models for medical and pharmacological applications, including compatibility with high‐throughput approaches.

## 2 Results

### 2.1 A Novel Skin Model: Self‐Assembled Skin Equivalents (SEs) With Monoclonal Knockout N/TERT‐1 Keratinocytes

**FIGURE 1:**
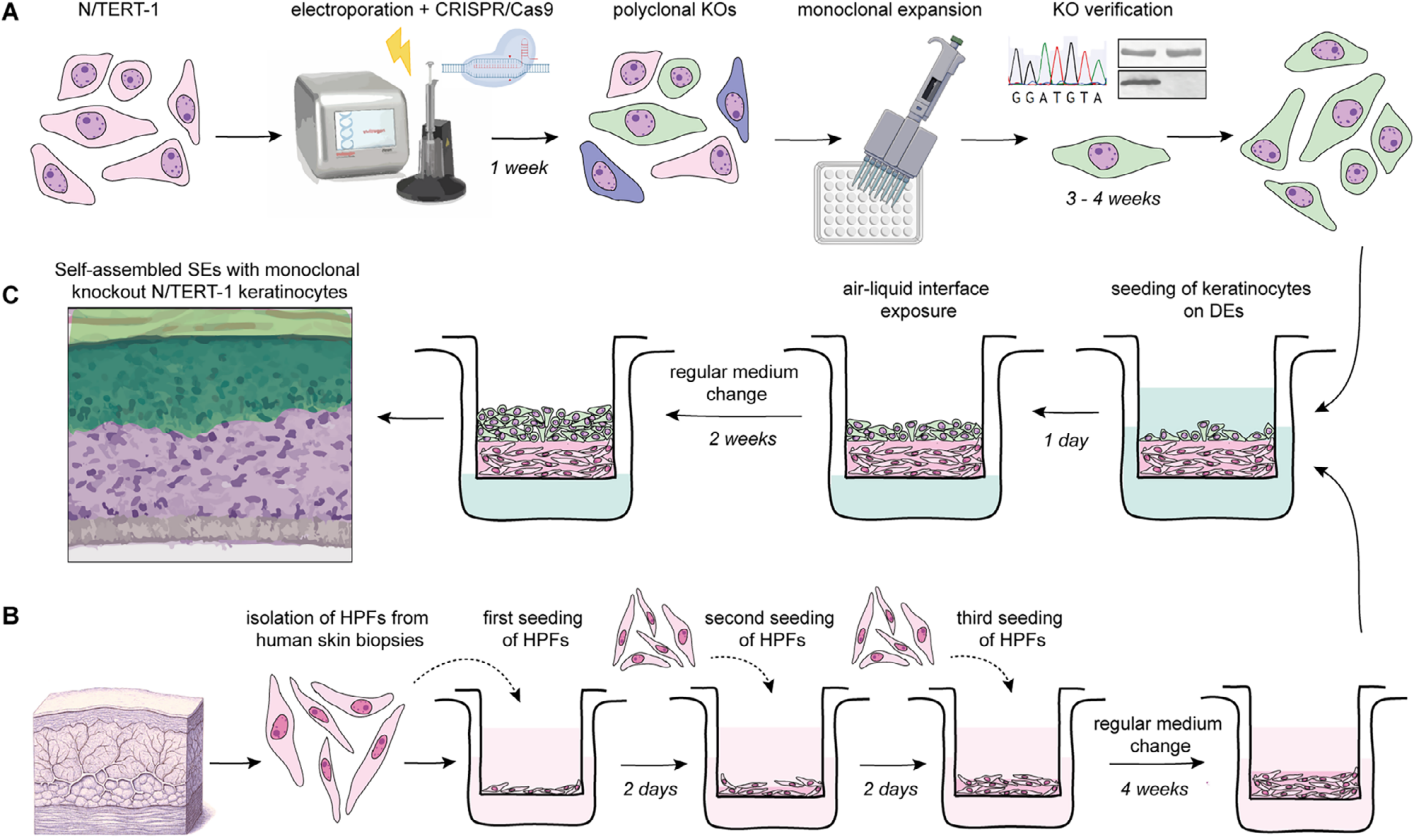
Scheme for the generation of a novel skin model based on monoclonal KO N/TERT‐1 keratinocytes and self‐assembled SEs. (A) Generation of monoclonal KO N/TERT‐1 keratinocytes. Electroporation of N/TERT‐1 keratinocytes with RNP complexes, consisting of gRNA and Cas9 protein, results in efficient and specific generation of KO cells with the CRISPR/Cas9 technique. Expansion of monoclonal populations from single cells allows the generation of homozygous KO cell populations, which are afterward verified for the absence of expression of the targeted gene(s) (sequencing and western blot). (B) Generation of DEs. HPFs isolated from human skin are seeded three times within 1 week on cell culture inserts for the generation of DEs and are kept in culture for 4 weeks to produce a self‐derived ECM. (C) SEs with monoclonal KO N/TERT‐1 keratinocytes. Monoclonal (or polyclonal) KO N/TERT‐1 keratinocytes are seeded onto DEs and, after 1 day, exposed to the air–liquid interface to induce their stratification, producing a fully differentiated epidermis after 2 weeks in culture.

SEs with a fibroblast‐derived ECM are compared to scaffold‐based SEs highly reproducible and exceptionally stable, although they require additional time investment in the production of the DEs (Figure 1) [31]. HPFs required for DEs production can be easily isolated in high numbers from human skin biopsies or are commercially available. To establish a model suitable for genetic manipulation, N/TERT‐1 keratinocytes were used for the epidermal component due to their capacity for long‐term expansion and clonal selection.

Monoclonal and polyclonal CRISPR/Cas9‐mediated KO N/TERT‐1 keratinocytes were generated by electroporation of Cas9‐gRNA RNP complexes. Monoclonal populations were obtained by single cell cloning and expanded prior to incorporation into SEs (Figure 1A). DEs were generated by repeated seeding of HPFs onto cell culture inserts, followed by prolonged culture to allow deposition of a fibroblast‐derived ECM (Figure 1B) [31]. Subsequently, wild‐type or CRISPR/Cas9‐modified N/TERT‐1 keratinocytes were seeded onto the DEs and cultured at the air‐liquid interface to induce epidermal stratification (Figure 1C).

This workflow enables the generation of scaffold‐free SEs incorporating genetically modified keratinocyte populations, including monoclonal KO cell lines.

### 2.2 Culture of N/TERT‐1 Keratinocytes in Self‐Assembled SEs

**FIGURE 2:**
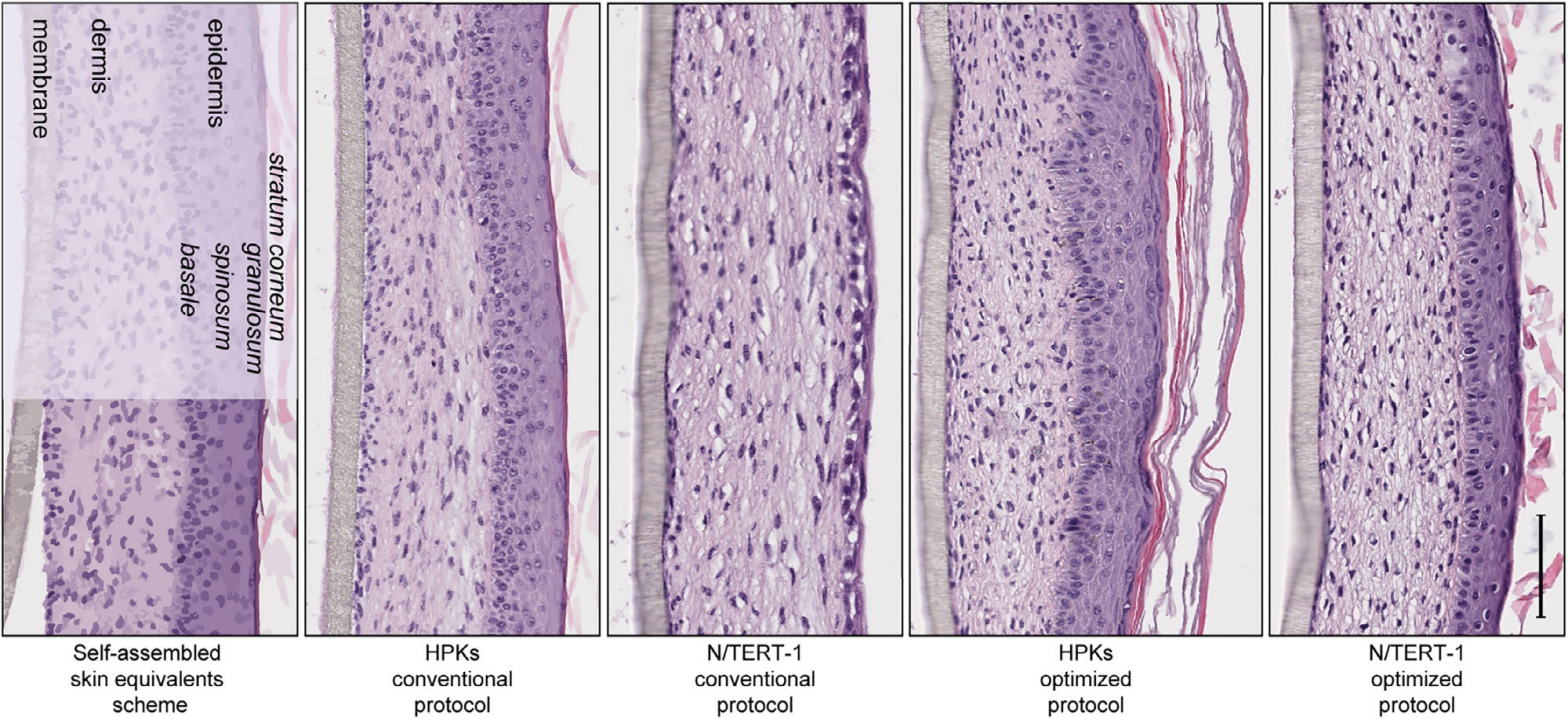
N/TERT‐1 keratinocytes form an epidermis on self‐assembled DEs. Wild‐type N/TERT‐1 keratinocytes were cultivated on DEs using both conventional (for SEs with HPKs) and optimized (for the N/TERT‐1 cell line) protocols. Histological analysis by hematoxylin and eosin (H&E) staining showed that, when the optimized protocol was followed, the SEs resembled the schematic representation of stratified skin. Scale bar: 100 µm.

The culture of wild‐type N/TERT‐1 keratinocytes in conventional HPK‐epidermal medium [31] on DEs results in a single keratinocyte layer without stratification or differentiation (Figure 2). To enable formation of an epidermis, culture conditions and the composition of the culture medium were modified, originating from formulations and co‐cultivation protocols used in published scaffold‐based SEs with N/TERT‐1 keratinocytes [50]. Using the optimized medium, N/TERT‐1 keratinocytes were seeded and cultivated on DEs, exposed to the air–liquid interface after 1 day, and cultured for 2 weeks prior to analysis.

Under these conditions, N/TERT‐1 keratinocytes formed a stratified epidermis, as confirmed by histological analysis (Figure 2). The resulting epidermis was thinner compared to that generated with HPKs but showed clear stratification and differentiation.

### 2.3 Generation of Monoclonal Knockout N/TERT‐1 Keratinocytes

**FIGURE 3:**
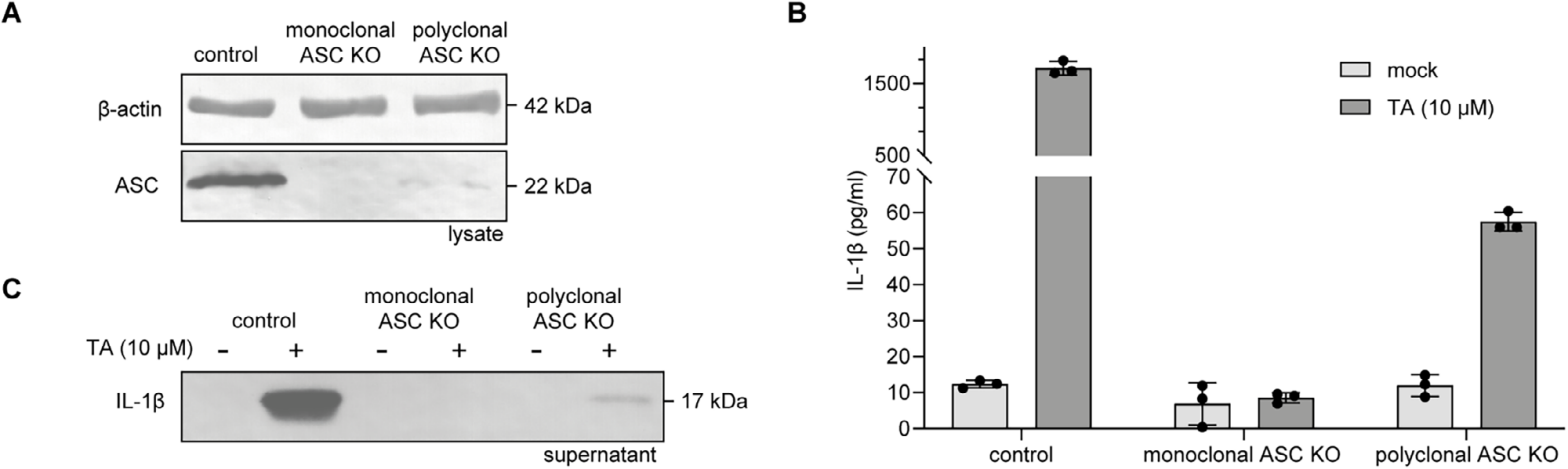
Characterization of monoclonal and polyclonal ASC KO N/TERT‐1 keratinocytes. After electroporation of N/TERT‐1 keratinocytes with RNP complexes targeting the *ASC* gene, the cells were either directly expanded as a bulk population or seeded for the generation of single‐cell clones, resulting in polyclonal or monoclonal ASC KO cell populations, respectively. (A) ASC protein expression of control, monoclonal ASC KO, and polyclonal ASC KO cells was determined by western blot. Expression of β‐actin served as a loading control. (B,C) Cells were mock‐treated or treated with 3 µm of the NLRP1 inflammasome activator talabostat (TA) and harvested after 24 h. Secretion of mature IL‐1β reflects inflammasome activation, which is dependent on expression of ASC, and was determined either by (B) enzELISA or (C) western blot. Each point is representative of a technical replicate from the same KO population. Data are shown as mean ± SD.

The protein apoptosis‐associated speck‐like protein containing a caspase recruitment domain (ASC) is essential for the activation of the NOD‐like receptor family pyrin domain‐containing 1 (NLRP1) inflammasome in human keratinocytes [51]. Upon assembly, inflammasomes induce activation and secretion of the pro‐inflammatory cytokines interleukin (IL)‐1β and ‐18 and secretion of other IL‐1 family proteins, such as IL‐1α or IL‐36γ [52, 53, 54, 55]. We used an established protocol for electroporation of HPKs with gRNA targeting the *ASC* gene to generate ASC KO N/TERT‐1 keratinocytes, with a non‐targeting gRNA serving as a control [45]. Then, we expanded the resulting cells either directly after KO generation or after single‐cell dilution for obtaining polyclonal or monoclonal cell populations, respectively. Monoclonal populations were additionally verified by checking for site‐specific indels in the *ASC* gene via Sanger sequencing (not shown), and western blot analysis revealed a robust decrease in ASC expression in polyclonal N/TERT‐1 KO keratinocytes and a complete loss of the protein expression in monoclonal N/TERT‐1 KO keratinocytes (Figure 3A).

To assess functional consequences, control and ASC KO cells were treated with talabostat, a strong activator of the NLRP1 inflammasome [56]. Whereas talabostat did not induce any secretion of IL‐1β in monoclonal ASC KO N/TERT‐1 cells, which completely lack expression of an active ASC protein, polyclonal KO cells released the cytokine, even though there was approximately a 30‐fold reduction compared to control cells (Figure 3B,C).

This experiment demonstrates that electroporation of N/TERT‐1 keratinocytes with RNP complexes yields a highly efficient KO that is indistinguishable at the protein level by western blot in both polyclonal and monoclonal populations. However, functional readouts revealed a small fraction of non‐targeted cells (less than 4%) within the polyclonal population. Although minor, this residual population can be sufficient to produce low‐level functional outputs, such as release of IL‐1 cytokines, which may propagate downstream signaling and ultimately confound the interpretation of pathway‐dependent readouts. These effects are avoided when using monoclonal KOs.

### 2.4 Culture of Monoclonal N/TERT‐1 Knockout Keratinocytes in SEs

**FIGURE 4:**
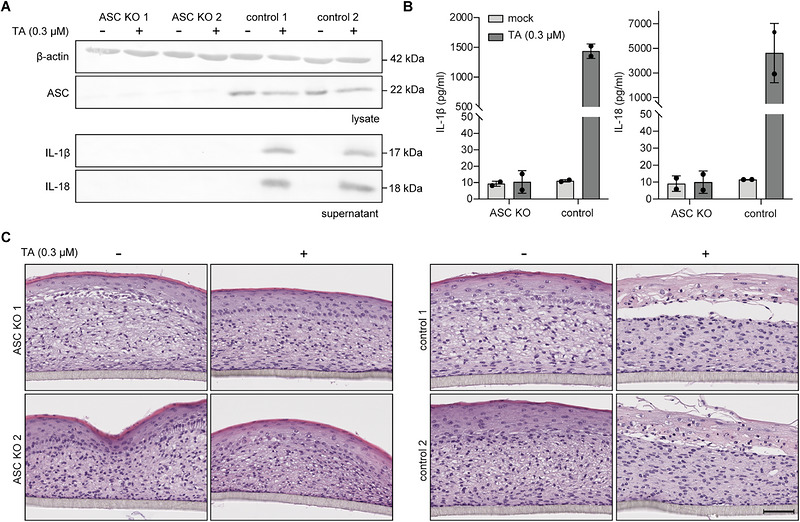
Characterization of SEs with monoclonal control or ASC KO N/TERT‐1 keratinocytes. SEs were generated with two KO populations (1 or 2), each, of monoclonal control or monoclonal ASC KO N/TERT‐1 keratinocytes, and either mock‐treated or treated with 0.3 µm of the NLRP1 activator talabostat (TA). SEs were harvested after 3 days. (A) The dermal and epidermal compartments of the SEs were separated and expression of the inflammasome protein ASC in keratinocytes in the epidermis was characterized by western blot, with the expression of β‐actin serving as a loading control. Proteins in the supernatants of SEs were precipitated using acetone. Maturation and secretion of IL‐1β and IL‐18 into the supernatants, reflecting ASC‐dependent inflammasome activation, were characterized by western blot. (B) Secretion of IL‐1β and IL‐18 is regulated by ASC‐dependent inflammasome activation in human keratinocytes and was analyzed by ELISA. (C) Histology of SEs with monoclonal control or ASC KO N/TERT‐1 keratinocytes following mock‐ or TA‐treatment was characterized by H&E staining. Each point represents an individual SE from the same KO population. Scale bar: 50 µm. Data are shown as mean ± SD.

Monoclonal control and ASC KO N/TERT‐1 keratinocytes were seeded onto DEs to assess their ability to form a stratified epidermis. Under optimized culture conditions, both control and ASC KO keratinocytes generated a differentiated epidermis (Figure 4C). To assess the functional consequences of the KO, SEs were treated for 3 days with talabostat, a specific pharmacological NLRP1 inflammasome activator in human skin [55]. After separation of the dermal and epidermal compartments, ASC expression was analyzed by western blot. ASC protein was absent in the epidermis of SEs generated from ASC KO keratinocytes (Figure 4A). Furthermore, talabostat‐induced secretion of the cytokines IL‐1β and IL‐18 into the cultivation medium of SEs was assessed either qualitatively by western blot (Figure 4A) or quantitatively by ELISA (Figure 4B). Most importantly, talabostat‐induced IL‐1β secretion was completely blocked in SEs generated from ASC KO keratinocytes.

This experiment demonstrates that monoclonal KO N/TERT‐1 keratinocytes, which completely lack ASC expression, can be cultured in SEs and form a differentiated epidermis that is unable to activate the NLRP1 inflammasome, reflected by the absence of secretion of pro‐inflammatory cytokines. Consequently, the histological alterations induced by talabostat in control SEs are absent in SEs generated from ASC‐deficient keratinocytes (Figure 4C).

**FIGURE 5:**
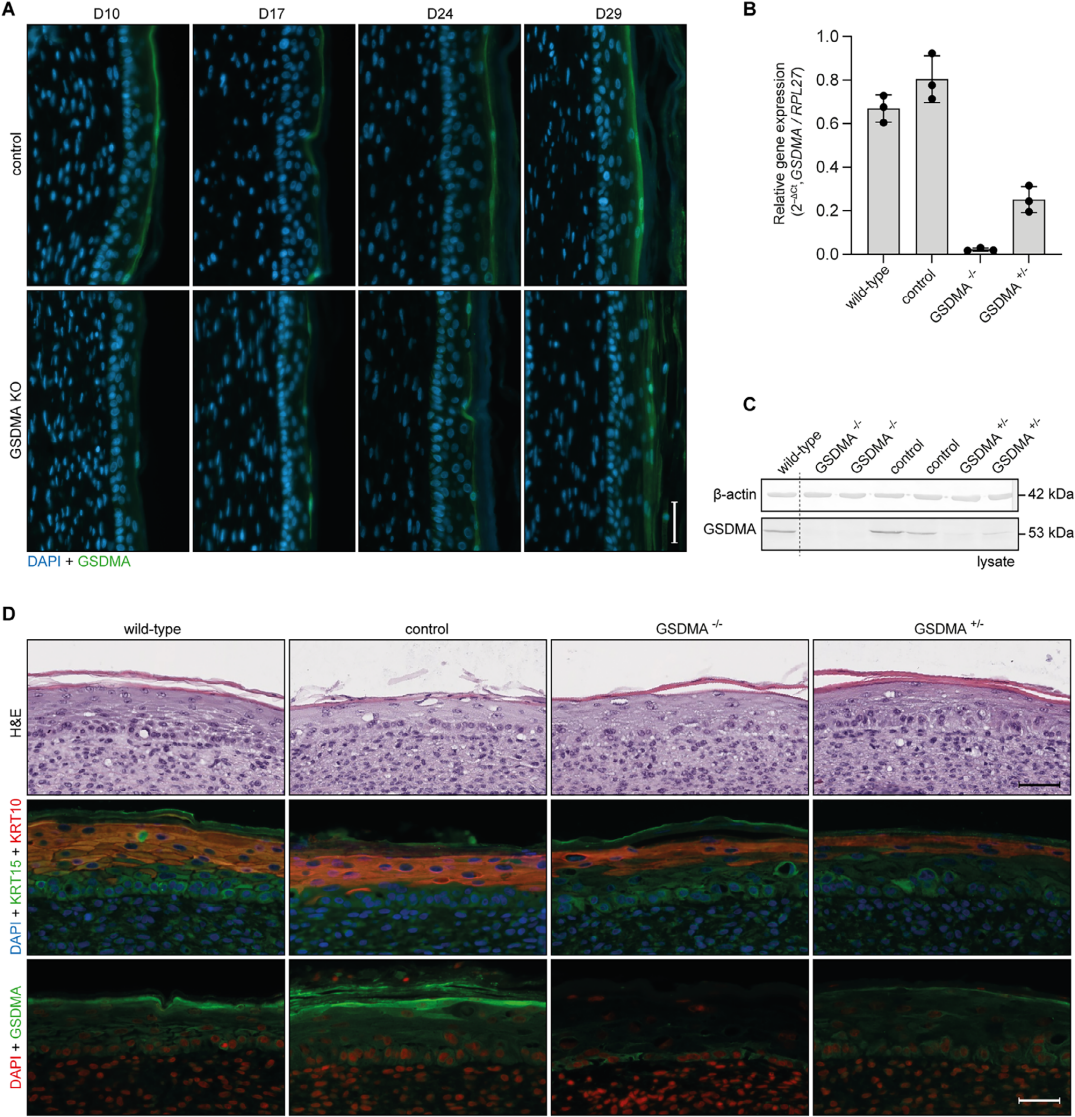
Characterization of SEs with monoclonal control, and homo‐ or heterozygous GSDMA KO N/TERT‐1 keratinocytes. (A) Histology of SEs, generated with polyclonal control or GSDMA KO HPKs and analyzed by IF after 10, 17, 24, and 29 days upon exposing them to the air‐liquid interface. Cells were stained for nuclei with DAPI (in blue) and for GSDMA (in green). (B–D) SEs were generated with monoclonal control, and homo‐ or heterozygous GSDMA KO N/TERT‐1 keratinocytes. (B) Dermis and epidermis were separated and expression of *GSDMA* in the epidermis was characterized by qPCR. (C) Dermis and epidermis were separated and expression of GSDMA protein in the epidermis was analyzed by western blot, with expression of β‐actin serving as a loading control. Lanes shown originate from the same gel/blot and were spliced for presentation. Splicing is indicated by a vertical dashed line. (D) Histology of SEs was analyzed by H&E staining and by IF. Cells were stained for nuclei with DAPI (in blue or red), and for keratin 10 (in red), keratin 15 (in green), or GSDMA (in green). Each point represents an individual SE grown from the same KO population. Scale bar: 50 µm. Data are shown as mean ± SD.

Furthermore, monoclonal gasdermin A (GSDMA) KO N/TERT‐1 keratinocytes were generated and seeded onto DEs. In contrast to ASC, which is ubiquitously expressed by human keratinocytes [51], expression of GSDMA is induced in keratinocytes when the cells reach advanced differentiation stages [57]. GSDMs represent a family of proteins, whose members are able to generate pores in cell membranes upon their proteolytic activation and are all expressed in human skin in keratinocytes, in part in a differentiation‐specific manner [58, 59]. GSDMA protein expression is not detectable in proliferating keratinocytes, such as in HPKs during standard monolayer culture, but is present in the *stratum granulosum* of stratified epidermis (Figure 5A–D). When SEs were generated from polyclonal GSDMA KO HPKs, GSDMA expression was still detectable in the stratum granulosum, although with a delayed onset (Figure 5A). Therefore, monoclonal GSDMA KO N/TERT‐1 keratinocytes were used for the further experiments. Upon culture of wild‐type or control KO N/TERT‐1 keratinocytes in SEs, GSDMA expression was strongly induced and could be detected in the epidermal compartment after its separation from the dermis, as determined by quantitative polymerase chain reaction (qPCR) (Figure 5B). In contrast, monoclonal GSDMA KO N/TERT‐1 keratinocytes showed absent or markedly reduced GSDMA mRNA expression (Figure 5B), the latter likely representing heterozygous GSDMA KO clones. Western blot analysis confirmed the absence of GSDMA protein in SEs generated from homozygous GSDMA^−/−^ cells and reduced levels in SEs derived from heterozygous GSDMA^+/−^ cells (Figure 5C). H&E staining revealed that all keratinocyte populations formed a stratified epidermis (Figure 5D). Immunofluorescence (IF) analysis confirmed GSDMA expression in differentiated keratinocytes in SEs generated from wild‐type and control cells, whereas expression was reduced or absent in SEs derived from GSDMA^+/−^ or GSDMA^−/−^ keratinocytes, respectively. Expression of keratin 15 was detected in the basal layer in all conditions, whereas keratin 10 expression appeared reduced in SEs generated from keratinocytes with low or absent GSDMA expression (Figure 5D).

These experiments demonstrate the utility of our model, particularly in experimental settings where complete gene depletion across the cell population is critical to addressing the underlying research questions. SEs are essential for studying the function of proteins that are expressed exclusively in differentiated keratinocytes, such as GSDMA. Furthermore, monoclonal KO keratinocytes are required to achieve complete ablation of expression of a given gene, which is feasible using indefinitely proliferating keratinocytes, such as the N/TERT‐1 cell line.

## 3 Discussion

Here we describe the establishment of a scaffold‐free full‐thickness SE model that combines a fibroblast‐derived dermal compartment with an epidermal layer based on CRISPR/Cas9‐modified poly‐ or monoclonal N/TERT‐1 keratinocytes.

This model builds on previously described self‐assembled DEs, in which fibroblasts generate their own ECM, resulting in improved structural and temporal stability and a closer resemblance to native dermal architecture compared to scaffold‐based systems [30, 31]. SEs reflect the compartmentation of human skin, the stratification of the epidermis and, therefore, are increasingly used as relevant skin models [25]. Because these models are still based on HPFs, they remain associated with donor‐specific variability. A replacement of HPFs in SEs by a commercially available cell line would help further standardize this model and would contribute to wider distribution and acceptance. Although their generation is more time‐consuming than scaffold‐based approaches, SEs incorporating a fibroblast‐derived ECM in the dermal compartment are qualitatively superior. They more closely recapitulate the in vivo ECM architecture [31], thereby supporting improved keratinocyte differentiation and *stratum corneum* formation [4], and, most importantly, exhibit greater stability, robustness, and reproducibility [4]. Particularly reproducibility is a prerequisite for paving the way to a widespread adaptation of the model by research as well as industry, including pharmaceutical or cosmetic companies.

A central feature of this model is the use of N/TERT‐1 keratinocytes, which combine the ability to form a differentiated epidermis with the capacity for long‐term expansion and clonal selection after genetic modification. While HPKs remain the gold standard for epidermal differentiation, their limited proliferative capacity restricts their use in applications requiring genetic manipulation. In contrast, immortalized keratinocytes such as N/TERT‐1 enable efficient generation of monoclonal CRISPR/Cas9‐edited cell lines, thereby facilitating precise genetic manipulation prior to incorporation into SEs allowing for addressing the function of genes or the establishment of disease models, such as for atopic dermatitis, psoriasis, or harlequin ichthyosis [49, 50, 55].

The present data demonstrate that N/TERT‐1 keratinocytes can form a stratified epidermis in scaffold‐free SEs under optimized culture conditions, extending previous studies that primarily used EEs or scaffold‐based systems [20, 22, 43]. The importance of monoclonal KO populations is highlighted by the functional differences observed between monoclonal and polyclonal ASC‐deficient keratinocytes. While polyclonal populations showed residual cytokine release upon inflammasome activation, monoclonal KO cells exhibited complete functional ablation. This indicates that even small fractions of non‐edited cells can influence pathway‐specific readouts and produce low‐level functional outputs and propagate downstream signaling, particularly in long‐term 3D cultures, where selective growth advantages may lead to overrepresentation of residual wild‐type cells. This also confirms that inflammasome signaling can be functionally recapitulated and selectively disrupted in the SE system. Given the limited conservation of the NLRP1 pathway between human and murine skin, such human‐based models provide an important platform for studying human‐specific inflammatory mechanisms [11, 60, 61]. Inflammasome activation in the SE model was induced using talabostat, the most specific activator of the NLRP1 inflammasome. Given the involvement of NLRP1 signaling in inflammatory skin diseases such as psoriasis, this system provides a relevant platform to investigate disease‐associated mechanisms [55].

In addition, if the protein product of the targeted gene is involved in proliferation or differentiation of keratinocytes, a KO might result in a selection advantage for the remaining wild‐type cells, and, therefore, could overgrow the KO cells, leading to a less reproducible and unstable model. Therefore, our novel model is suitable for investigating proteins that are expressed predominantly in differentiated keratinocytes. GSDMA represents such a case, as its expression is induced during epidermal differentiation. Complete ablation of GSDMA expression was achieved only in monoclonal KO populations, whereas polyclonal approaches resulted in residual expression. Notably, reduced expression of the differentiation marker keratin 10 in GSDMA‐deficient SEs suggests a potential role of GSDMA in keratinocyte differentiation, consistent with recent findings in murine models [62]. These observations highlight the value of the system for studying differentiation‐dependent gene function in a 3D environment.

In the past, gRNA and Cas9 protein were delivered upon lentiviral transduction, which can potentially lead to unspecific targeting effects due to the permanent expression of gRNA and Cas9. Consequently, the properties of monoclonal cell populations varied strongly due to these side effects, depending on virus copy numbers integrated into the target cells and the length of their cultivation. In contrast, delivery of RNP complexes of Cas9 and gRNA by electroporation avoids these problems and no major differences between different monoclonal populations of the same KOs were observed in our experiments [39, 43, 44, 45, 47].

Compared to scaffold‐based SEs, the scaffold‐free approach provides several advantages, including reduced matrix contraction, increased structural stability and extended culture duration, enabling long‐term experiments and sustained epidermal regeneration [7, 27]. However, some limitations remain. The dermal compartment still relies on HPFs, introducing donor variability, and N/TERT‐1 keratinocytes, although more standardized, may not fully recapitulate all aspects of HPK biology. Future developments may include the use of standardized fibroblast cell lines and further incorporation of additional skin components, such as immune cells, vascular structures, or microbiome elements, to increase physiological complexity.

In conclusion, scaffold‐free SEs incorporating monoclonal CRISPR/Cas9‐edited N/TERT‐1 keratinocytes represent a robust, reproducible, and versatile platform for studying gene function in human skin. This system enables precise genetic manipulation in a physiologically relevant 3D context and provides a valuable tool for mechanistic studies, disease modeling, and pharmacological applications.

## 4 Materials and Methods

### 4.1 Human Skin Samples

Skin sampling was conducted according to the principles of the Declaration of Helsinki. Skin samples were obtained from surgical excisions not required for histological diagnostics in Department of Dermatology, University Hospital Zurich or Kinderchirurgie und Familienmedizin Doktorhaus AG Fällanden. Patients signed informed consent, following approval from the Kantonale Ethikkommission (KEK) (KEK‐ZH‐Nr.2015‐0198 and BASEC‐Nr.2024‐01030). Trunk skin samples were used for the isolation of HPFs, and prepuce skin samples were used for the isolation of HPKs.

### 4.2 Cell Culture in 2D

HPKs and HPFs were isolated as previously described [46, 47]. HPKs and N/TERT‐1 keratinocytes, the latter of which were obtained from the Rheinwald lab [20], were grown in keratinocyte serum‐free medium supplemented with epidermal growth factor (EGF) and bovine pituitary extract (BPE) (17005042, Gibco). For passaging and seeding for experiments or on DEs, keratinocytes were detached with TrypLE Express Enzyme (1X), phenol red (12605010, Gibco). HPFs were isolated from the dermis of human trunk skin samples after incubation with a solution of 1 mg/mL collagenase (C2674, Sigma) and 0.05 mm CaCl_2_ in phosphate buffered saline (14190144, Gibco) for 2 h at 37°C. A single‐cell suspension was obtained by pipetting the dermis up and down in DMEM, high glucose, pyruvate (41966052, Thermo Fisher Scientific), containing 25% FBS (S181H, BioWest) and 1% antibiotic/antimycotic (15240062, Thermo Fisher Scientific). HPFs were cultivated in DMEM supplemented with 10% FBS (S181H, BioWest) and 1% antibiotic/antimycotic (15240062, Thermo Fisher Scientific). Mycoplasma contamination was regularly examined. All cells and skin equivalents were kept at 37°C, 5% CO_2_, and 20% O_2_. The culture medium was changed every 2–3 days. N/TERT‐1 keratinocytes cultivated in monolayer were treated 48 h after their seeding with 10 µm talabostat (HY‐13233A, Lucerna‐Chem).

### 4.3 Generation of CRISPR/Cas9‐Mediated Knockout Keratinocytes by Electroporation

CRISPR/Cas9‐mediated gene KO with HPK or N/TERT‐1 populations was performed as described previously [45]. Briefly, RNP complexes were prepared by mixing TrueCut HiFi Cas9 Protein (5 µg/µL, A50577, Invitrogen) with custom‐designed TrueGuide Synthetic gRNA (1.5 nmol, A35534, Invitrogen) in a 1:1 molar ratio. gRNAs without the PAM sequence (ASC gRNA—CAUGUCGCGCAGCACGUUAG, GSDMA gRNA—GGAUGUACCAAAGACGGUGA) were designed using the free online software ChopChop [63] with the latest human genome annotation (Homo Sapiens hg38/GRCh38). For both KOs, the TrueGuide sgRNA Negative Control, non‐targeting 1 (A35526, Invitrogen) was used as a non‐targeting control. The mixtures were incubated for 20 min at room temperature. Cell pellets of 0.15 × 10^6^ HPKs or N/TERT‐1 keratinocytes were resuspended in 5 µL of electroporation buffer R, included in the Neon Transfection System 10 µL Kit (MPK1096, Invitrogen), and then transferred to the 5 µL of RNP mix. Electroporation was performed according to the manufacturer's protocol and carried out at 1700 V, 20 ms, 1 pulse, with the Neon Transfection System (MPK5000, Invitrogen). Electroporated cells were directly seeded into flasks and cultured for a week before further experiments or seeding on top of DEs. KO efficiencies were assessed at the protein level by western blot. For preparation of monoclonal populations, electroporated keratinocytes were diluted to a concentration of 0.5 cells/well and seeded in 96‐well plates. Monoclonal populations were cultivated for ≈3 weeks and characterized by Sanger sequencing (Microsynth AG) of the targeted region of the *ASC* or *GSDMA* gene and by western blot analysis for the absence of ASC or GSDMA protein (Figure 1).

### 4.4 Generation of Scaffold‐Free Dermal Equivalents from HPFs

DEs were generated as described [31]. Briefly, HPFs of passage 5 were expanded for 2 weeks by two passages in DMEM supplemented with 10% FBS (F7524, Sigma) and 1% antibiotic/antimycotic (15240062, Thermo Fisher Scientific). 0.5 × 10^6^ HPFs were seeded on day 1, 3, and 5, in 1 mL of dermal cultivation medium and onto 12‐well translucent ThinCerts with high‐density 0.4 µm pores (11.31 cm^2^ culture area, 392‐0054, Avantor), placed in deep‐well plates (665110, Greiner Bio‐One) with 5 mL of dermal cultivation medium and incubated at 37°C, 5% CO_2_, and 20% O_2_ over a total culture time of 4 weeks. Dermal cultivation medium was composed of 3:1 DMEM/Ham's F12 (41966052/ 21765‐029, Thermo Fisher Scientific) containing 10% FBS (F7524, Sigma) and 1% antibiotics/antimycotics (15240062, Thermo Fisher Scientific), 200 µg/mL 2‐phospho‐L‐ascorbic acid (49752, Sigma), 1 ng/mL TGFβ‐1 (100‐21, PeproTech), 2.5 ng/mL EGF (PHG0311, Invitrogen), 5 ng/mL FGF‐basic (100‐18B, PeproTech), and 5 µg/mL insulin (I6634, Sigma). Medium was changed every other day in both compartments: the insert (1 mL) and the well below the insert (5 mL) (Figure 1).

### 4.5 Generation of Scaffold‐Free Skin Equivalents with HPKs

SEs with HPKs were generated as described [31]. Briefly, 1 day before seeding of the keratinocytes, dermal medium in both compartments (insert and well) was exchanged to HPK‐epidermal medium, composed of 3:1 DMEM/Ham's F12 (41966052/ 21765‐029, Thermo Fisher Scientific) containing 10% FBS (F7524, Sigma) and 1% antibiotics/antimycotics (15240062, Thermo Fisher Scientific), 200 µg/mL 2‐phospho‐L‐ascorbic acid (49752, Sigma), 0.4 µg/mL hydrocortisone (H0888, Sigma), and 0.1 nm cholera toxin (C8052, Sigma). Next day, 0.25 × 10^6^ HPKs, wild‐type or CRISPR/Cas9‐modified, were seeded per DE in 1 mL of HPK‐epidermal medium. With the seeding of HPKs, the medium in the well was also exchanged (5 mL). After 3 days of submersed growth without a medium change, the epithelial co‐cultures were air‐lifted (permanent removal of medium from the insert) and the HPK‐epidermal medium within the wells (4 mL) was changed every other day until the SEs were harvested for analysis (2 weeks) (Figure 1).

### 4.6 Generation of Scaffold‐Free Skin Equivalents With N/TERT‐1 Keratinocytes

SEs with N/TERT‐1 keratinocytes were generated using the optimized protocol. 1 day before seeding the keratinocytes, dermal medium in both compartments (insert and well) was exchanged to N/TERT‐1‐epidermal medium, composed of DMEM/Ham's F12 (11320074, Thermo Fisher Scientific) containing 10% FBS (F7524, Sigma) and 1% antibiotics/antimycotics (15240062, Thermo Fisher Scientific), 0.4 µg/mL hydrocortisone (H0888, Sigma), 10 ng/mL EGF (PHG0311, Invitrogen), 5 µg/mL insulin (I6634, Sigma), 5 µg/mL apo‐transferrin (T1147, Sigma), 20 pm 3,3′,5‐Triiodo‐L‐thyronine (T6397, Sigma), and 0.1 nm cholera toxin (C8052, Sigma). On the next day, 0.5 × 10^6^ N/TERT‐1 keratinocytes, wild‐type or CRISPR/Cas9‐modified KO monoclones, were seeded per DE in 1 mL of N/TERT‐1‐epidermal medium. With the seeding of N/TERT‐1 keratinocytes, the medium in the well was also exchanged (5 mL). After overnight submersed growth, the epithelial co‐cultures were air‐lifted (permanent removal of medium from the insert) and the N/TERT‐1‐epidermal medium in the wells (4 mL) was changed every other day, throughout 1 week. During the second week of medium change, the N/TERT‐1‐epidermal medium was supplemented with 50 µg/mL 2‐phospho‐L‐ascorbic acid (49752, Sigma) (4 mL) and changed every other day until the SEs were harvested for analysis (2 weeks) (Figure 1).

### 4.7 Treatment of Skin Equivalents

SEs with monoclonal CRISPR/Cas9‐modified KO N/TERT‐1 keratinocytes were either mock‐treated or treated with talabostat. Medium in wells of 2‐week‐old air‐lifted SEs was exchanged to Opti‐MEM (11058021, Thermo Fisher Scientific), supplemented with 0.3 µm talabostat (HY‐13233A, Lucerna‐Chem) or DMSO vehicle (D8418, Sigma) for mock‐treatment.

### 4.8 SDS‐PAGE and Western Blot Analysis

Epidermis of SEs was collected separately upon dispase II digestion for 30 min at 37°C (04942078001, Roche) and homogenized using the TissueLyser II (5 min, 25/s Hz, QIAGEN) in buffer containing 4% SDS and 100 mm Tris HCl pH 7.6. Protein concentration was determined using Pierce BCA Protein Assay Kit (23227, Thermo Fisher Scientific). Absorbance was measured using the Cytation 3 Cell Imaging Reader (Agilent Technologies). Proteins in solution were then incubated at 95°C for 5 min, sonicated and separated from the pellet upon centrifugation (16.000 rcf, 5 min). Before loading into the gels, proteins were diluted 1:5 in loading buffer, containing glycerol with 1% bromophenol blue. Monolayer cell lysates were harvested directly in protein lysis buffer, containing 4% SDS, 100 mm Tris HCl pH 7.6, 40% glycerol, and 1% bromophenol blue, boiled at 95°C for 5 min, sonicated and separated from the cell debris upon centrifugation (16.000 rcf, 5 min). Cell culture supernatants were precipitated in 2.5X volumes of acetone (179124, Sigma) by overnight incubation at −20°C, followed by centrifugation for approximately 2 h (4000 rcf at 4°C), and resuspension in protein lysis buffer. Proteins were separated by SDS‐PAGE and analyzed by immunoblotting (ACTB: A544, Sigma; ASC: AL177, Adipogen; IL‐1β: MAB201, R&D Systems; IL‐18: PM014, MBL; GSDMA: 49307S, Cell Signaling Technology). Protein bands were detected using BCIP/NBT Color Development Substrate (S3771, Promega) according to the manufacturer's instructions. Bound antibodies were visualized by colorimetric reaction, and signal intensity was recorded using standard imaging equipment.

### 4.9 Real‐Time PCR

Epidermis of SEs was collected separately upon dispase II digestion for 30 min at 37°C (04942078001, Roche), homogenized using the TissueLyser II (5 min, 25/s Hz, QIAGEN) in TRI Reagent (T9424, Sigma), and total RNA was isolated according to the manufacturers’ instructions. cDNA from mRNA was synthesized using the RevertAid First Strand cDNA Synthesis Kit (K1622, Thermo Fisher Scientific) and Oligo (dT)_18_ Primers (SO132, Thermo Fisher Scientific). mRNA expression levels were determined by quantitative real‐time PCR, using the LightCycler 480 (Roche) instrument and FastStart Essential DNA Green Master (06924204001, Roche) with specific primer pairs for *GSDMA* (TACGTCCGCACCGACTACA, CAGAGTGCTGTTCTGCGAGA). mRNA levels were normalized to the housekeeper *RPL27* (ATCGCCAAGAGATCAAAGATAA, TCTGAAGACATCCTTATTGACG).

### 4.10 Histology

SE samples were fixed overnight in 4% formalin, dehydrated automatically, and embedded in paraffin (Leica EG 1150, Leica microsystem). Paraffin blocks were cut into 5 µm‐thick sections and stained with H&E automatically or analyzed by IF.

### 4.11 Immunofluorescence

Paraffin sections were deparaffinized in xylene, rehydrated by 5 min incubation, each, in decreasing concentrations of ethanol (100%, 96%, 80%, 70%, 50%, and 0% in ddH_2_O), and heated in a steamer for 30 min in antigen retrieval solution at a pH of either 9 or 6, depending on the antibody (s236784‐2/S1699, both Dako). Sections were then blocked for 2 h at room temperature with 10% bovine serum albumin in DPBS (14190094, Gibco) and incubated over night at 4°C with the primary antibody (KRT10: 905404, BioLegend; KRT15: GP‐CK15, Progen, GSDMA: 49307S, Cell Signaling Technology), diluted 1:100 in 5% bovine serum albumin (A9647, Sigma) in DPBS (14190094, Gibco). On the next day, sections were washed three times in DPBS‐T, with 0.05% Tween 20 (P2287, Sigma) in DPBS, and incubated with the secondary antibody (Goat Anti‐Rabbit IgG + DyLight 488: ab96883, abcam; Goat anti‐Rabbit IgG + Alexa Fluor 647: A‐21246, Invitrogen; Goat Anti‐Guinea pig IgG + Alexa Fluor 488: ab150185, abcam) diluted 1:200 in 5% bovine serum albumin in DPBS for 1.5 h at room temperature. Then, the secondary antibody was removed and DAPI staining (D9542, Sigma), diluted 1:1000 in 5% bovine serum albumin in DPBS, was applied for 5 min at room temperature. After 3 washes in DPBS‐T, the coverslips were mounted on glass slides using ProLong Gold Antifade Mountant (P36934, Thermo Fisher Scientific). Tissues were imaged by fluorescence microscopy (Zeiss Axiocam 503 mono).

### 4.12 ELISA Assay

Cell culture supernatants were collected, cleared by centrifugation (400 rcf, 5 min), and the release of human IL‐1β and IL‐18 was measured by ELISA (DY201 and DY318, both R&D Systems), according to the manufacturer's instructions. Absorbance was measured using the Cytation 3 Cell Imaging Reader (Agilent Technologies).

## Conflicts of Interest

The authors declare no conflicts of interest.

## Data Availability

The data that support the findings of this study are available from the corresponding author upon reasonable request.
